# A hard X-ray nanoprobe beamline for nanoscale microscopy

**DOI:** 10.1107/S0909049512036783

**Published:** 2012-09-05

**Authors:** Robert P. Winarski, Martin V. Holt, Volker Rose, Peter Fuesz, Dean Carbaugh, Christa Benson, Deming Shu, David Kline, G. Brian Stephenson, Ian McNulty, Jörg Maser

**Affiliations:** a Center for Nanoscale Materials, Argonne National Laboratory, 9700 South Cass Avenue, Argonne, IL 60441, USA; b Advanced Photon Source and Center for Nanoscale Materials, Argonne National Laboratory, 9700 South Cass Avenue, Argonne, IL 60441, USA; c Advanced Photon Source, Argonne National Laboratory, 9700 South Cass Avenue, Argonne, IL 60441, USA

**Keywords:** X-ray nanoprobe, X-ray microscopy, zone plate, X-ray fluorescence, nanodiffraction, nanotomography

## Abstract

The Hard X-ray Nanoprobe Beamline (or Nanoprobe Beamline) is an X-ray microscopy facility incorporating diffraction, fluorescence and full-field imaging capabilities designed and operated by the Center for Nanoscale Materials and the Advanced Photon Source at Sector 26 of the Advanced Photon Source at Argonne National Laboratory. This facility was constructed to probe the nanoscale structure of biological, environmental and material sciences samples. The beamline provides intense focused X-rays to the Hard X-ray Nanoprobe (or Nanoprobe) which incorporates Fresnel zone plate optics and a precision laser sensing and control system. The beamline operates over X-ray energies from 3 to 30 keV, enabling studies of most elements in the periodic table, with a particular emphasis on imaging transition metals.

## Abstract 2

The Hard X-ray Nanoprobe Beamline is a precision platform for scanning probe and full-field microscopy with 3–30 keV X-rays. A combination of high-stability X-ray optics and precision motion sensing and control enables detailed studies of the internal features of samples with resolutions approaching 30 nm.

## 1. Introduction

In the last few decades the technology and methods developed for synchrotron X-ray research have contributed significantly to our understanding of structural biology (Doyle *et al.*, 1998 ▶; Schotte *et al.*, 2003 ▶), materials science (Srajer *et al.*, 2006 ▶; Poulsen *et al.*, 2001 ▶; Banhart *et al.*, 2001 ▶) and condensed-matter physics (Reichert *et al.*, 2000 ▶; Alagna *et al.*, 1998 ▶). Hard X-ray microscopy and imaging have made major additions to our knowledge in these areas, but until recently were typically limited to spatial resolutions of only hundreds of nanometers (Kirz & Jacobsen, 2009 ▶). The Nanoprobe Beamline was designed to enable hard X-ray microscopy with unprecedented spatial resolution. The unique capabilities (high stability, high flux, high beam coherence) of the beamline provide for the operation of a state-of-the-art microscope, the Hard X-ray Nanoprobe (or Nano­probe). A detailed description of the design and performance of the Hard X-ray Nanoprobe is provided in an additional publication (Winarski *et al.*, 2012 ▶). Additionally, these capabilities facilitate other pioneering developments that focus on highest-resolution X-ray microscopy (Yan *et al.*, 2011 ▶; Cummings *et al.*, 2012 ▶; Rose *et al.*, 2011 ▶).

**Figure 1:**
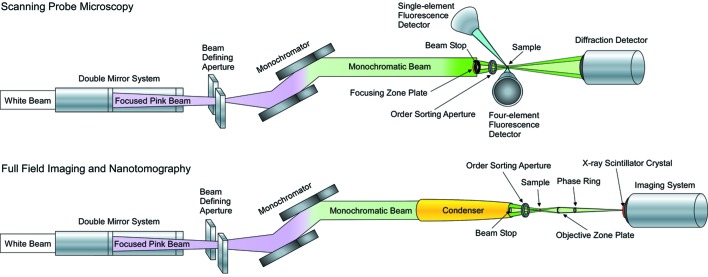
Optical schematic of the Nanoprobe Beamline showing the two modes of operation. For scanning probe microscopy the mirror system focuses the X-rays onto an aperture which defines the horizontal size of the beam. The monochromatic beam is then focused by a zone plate onto the sample. For full-field imaging and nanotomography the aperture is opened and the monochromatic beam is focused by an elliptical condenser onto the sample.

The Nanoprobe Beamline, shown schematically in Fig. 1 ▶, is a precision facility for scanning probe and full-field microscopy with 3–30 keV X-rays (Maser *et al.*, 2005 ▶, 2006 ▶). A combination of nanofocusing X-ray optics and precision motion sensing and control enables detailed studies of the internal features of samples with a resolution approaching 30 nm. To maximize the X-ray flux from the Fresnel zone plate optics that are used in focusing and detecting the X-rays in the Nanoprobe, the beamline was designed to accommodate the thermal power and power densities associated with illumination from two undulators in series. This placed unique design requirements on the optical components for the beamline.

The Nanoprobe utilizes three different techniques for probing the nanoscale structure of samples: X-ray fluorescence, X-ray diffraction and full-field X-ray imaging (which, combined with a precision rotation stage, can also yield nanoscale tomographic images) (Sayre & Chapman, 1995 ▶). The precision optics manipulation necessary for imaging at a resolution approaching 30 nm required the development of unique sensing and motion controls to ensure accurate detection of features inside of samples. The Nanoprobe optics and controls platforms were designed to achieve a spatial resolution of 10 nm with the future development of higher-resolution zone plates.

## 2. Beamline X-ray optics

**Figure 2:**
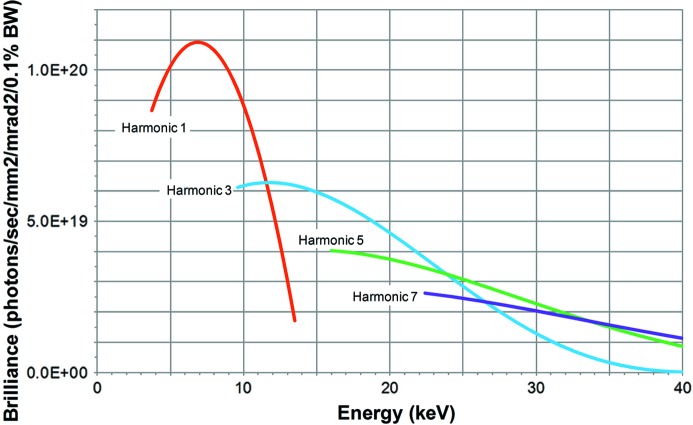
Tuning curves for two phased APS type-A undulators. Undulator brilliance calculated for these parameters: APS storage ring operating at a beam energy of 7.0 GeV, 100 mA ring current, 2.51 nm rad low-emittance lattice, 1.5% coupling, minimum undulator gap of 11.0 mm, two 2.4 m-long undulators, with phasing factors of 0.75 (harmonic 1) and 0.90 (harmonic 3).

The Nanoprobe Beamline utilizes two APS type-A undulators (Cai *et al.*, 1996 ▶), each with 3.3 cm periodic spacing and 2.4 m in length, for a total length of 4.8 m, to cover the energy spectrum from 3 to 30 keV, as shown in Fig. 2 ▶. For the current APS operating parameters of 7.0 GeV and 100 mA stored beam current, each undulator provides up to 5.7 kW of X-ray power. When operated in phase, the brightness of the two undulators together is 2.7 times that of a single undulator (30% increase over two unphased type-A undulators), with an angular power density of up to 19.2 kW mrad^−2^ (Ilinski, 1998 ▶; Dejus *et al.*, 1994 ▶).

**Figure 3:**
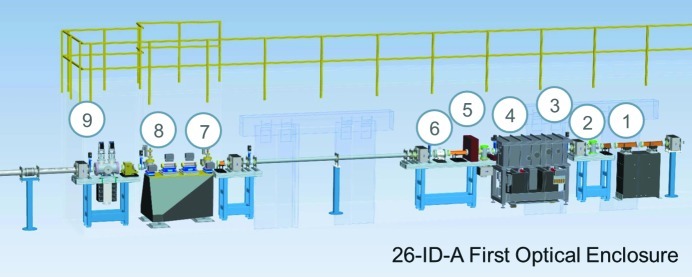
Layout of X-ray optical components in 26-ID-A: (1) white beam slits, (2) mask M26-A1, (3) beam visualization monitor, (4) mirror system, (5) beam visualization monitor, (6) mask M26-A2, (7) mask M26-A3, (8) beam defining aperture, (9) integral shutter assembly.

The beamline’s first optical enclosure, designated 26-ID-A, shown in Fig. 3 ▶, contains beam size defining slits, collimators, mirrors, and masks that are used to remove the incoherent portion of the X-ray beam which cannot be focused to a diffraction-limited spot. The raw ‘white’ beam produced by the undulators is directed onto the first set of beam slits, designated as white beam slits (WBS), which determine the size of the X-ray beam that will be delivered onto the mirror system (Benson *et al.*, 2007 ▶). The WBS consist of two grazing-incidence Glidcop (North American Höganäs) water-cooled bodies that intercept the X-ray beam along their full lengths and shape the beam with tungsten blades located at the upstream ends of the slit bodies. Directly behind the WBS there is a mask and beam visualization monitor. The mask (M26-A1) is in place to encompass any possible beam steering errors from the storage ring that could damage optical components located further down the beamline. The beam visualization monitor (FMB GmbH fluorescence monitor) consists of an angled diamond screen that can be lowered into the beam and a video camera that captures an image of the fluorescence caused by the X-ray beam as it passes through the screen. This monitor allows for a direct image of the shaping of the X-ray beam determined by adjusting the opening size of the WBS.

The X-ray beam is directed onto the surface of the first mirror in the water-cooled double-mirror system (Accel Instruments GmbH Model 1579 Double Mirror System) located 30 m from the X-ray source inside the APS storage ring. The first mirror, designated M1a, is a 464 mm (380 mm cooled active area) long, 60 mm wide X-ray mirror which can focus the diverging incident beam *via* a bender mechanism with a cylindrical bend profile, adjustable from flat to a 2.5 km radius of curvature (demagnifying ratio of 3 to 1). The beam reflected from M1a is directed onto a flat second mirror, M1b, which is 1000 mm (900 mm cooled active area) in length by 70 mm wide, which directs the beam down the beamline. The two mirrors maintain a 4.8 mm horizontal offset from the direct beam trajectory delivered by the APS storage ring. The mirrors are polished single-crystal silicon substrates (roughness of less than 0.2 nm) that provide for harmonic rejection by directing the X-ray beam onto striped coatings (Si, Cr and Pt) and varying the rotation angle of the mirrors. Because of the grazing-incidence geometry and the long illuminated length of the mirrors, the system can be thermally stabilized (cooled) with water. The heat distribution along the mirror surfaces does not vary by more than 0.1 K even at the highest heat load as determined by resistance temperature detectors located along the mirror lengths. The mirrors incorporate side-clamped cooling plates to remove heat, rather than internal channels, which could introduce cavitation vibrations. Filtering out the higher energy portions of the undulator spectrum reduces the heat load on downstream components and removes possible signal noise that could affect experiments.

The X-ray beam from the mirror systems (now known as pink beam) is directed through a second beam visualization monitor for tracking beam steering from the mirrors, through additional masks and onto the beam defining aperture (BDA) which is located 40 m from the X-ray source inside the APS storage ring. The X-ray beam source provided by the undulator source has a vertically small but horizontally large size (10 µm by 280 µm). The BDA consists of a series of precision slits mounted on a low-vibration mount, which are used to create a sharply defined secondary source with a horizontal size ranging from 20 µm to 2 mm for illuminating the remaining beamline and Nanoprobe optics. The X-ray beam then passes through an integral shutter assembly which can absorb the power of the X-ray beam as well as block all radiation from travelling outside the experimental station, and through an evacuated transport line to the second experimental station.

**Figure 4:**
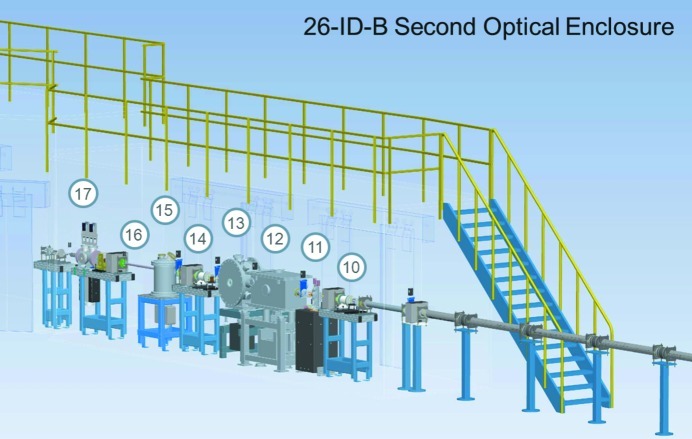
Layout of X-ray optical components in 26-ID-B: (10) mask M26-B1, (11) pink beam slits, (12) double-multilayer monochromator, (13) double-crystal monochromator, (14) mask M26-B2, (15) polarizer, (16) beam chopper, (17) integral shutter assembly.

The second optical enclosure, designated 26-ID-B, shown in Fig. 4 ▶, contains additional beam size defining slits, collimators, monochromators, space for a polarizer, a beam chopper, and masks that are used to further remove the incoherent portion of the X-ray beam. The X-ray beam from the 26-ID-A station (pink beam) travels through the transport section and is directed through a protective mask and onto another set of beam slits, designated as pink beam slits (PBS) (Benson *et al.*, 2007 ▶), which determine the size of the X-ray beam that will be delivered onto the monochromator crystals. The PBS are identical in design to the WBS, but are smaller in length as they dissipate a smaller thermal load.

The beamline utilizes two monochromators: a double-multilayer monochromator (DMM) is located behind the PBS, followed by a double-crystal monochromator (DCM). The DMM (Oxford Danfysik Model S1623), located 62 m from the X-ray source, is a high-intensity low-energy-resolution (Δ*E*/*E* = 0.01) monochromator that uses water-cooled W/Si multilayers (2.5 nm bilayer spacing) that can provide X-rays for full-field imaging experiments (Chu *et al.*, 2002 ▶). The DCM (Oxford Danfysik Model S1614), located 63.2 m from the X-ray source, is used for high-energy-resolution imaging (Δ*E*/*E* = 1.7 × 10^−4^) (Batterman & Cole, 1964 ▶). The DCM is a liquid-nitrogen (LN_2_) cooled Si (111) double-crystal monochromator that has been designed to minimize thermal distortion of the crystals, thereby preserving the wavefront quality on the nanofocusing optics. The LN_2_ cooling unit (Oxford Danfysik Model S1689 Cryocooler Series D) has been specially tuned with the monochromator in order to reduce vibrations owing to coolant flow. The crystals are vertically offset by 5.6 mm and have rigid crystal mounts to limit possible system mechanical vibrations. We currently operate in a range of energies between 7 and 12 keV. We have measured a monochromatic beam flux density of 2.5 × 10^13^ photons s^−1^ mm^−2^ (0.01% bandwidth)^−1^ (1 mm × 1 mm slits at 72 m from the X-ray source with 9.75 keV photons). Angular stability has been measured to be 0.657 µrad peak to peak (0.232 µrad RMS).

Space downstream from the DCM is reserved for an X-ray crystal polarizer which will be used to convert the now monochromatic linearly polarized X-rays into circularly polarized X-rays by passing the beam through a thin diamond crystal oriented near a Bragg reflection (Freeland *et al.*, 2002 ▶; Lang & Srajer, 1995 ▶). The wavefields inside the crystal propagate at different phase velocities for different linear polarization directions (changing the angle from the Bragg condition changes the polarization). Circularly polarized X-rays will be used to probe nanoscale magnetic structure using dichroism methods (Schütz *et al.*, 1987 ▶).

Behind the polarizer is a high-speed beam chopper which can be configured to select individual ∼100 ps pulses from the storage ring (during special asymmetric fill modes) for time-resolved studies (Gembicky *et al.*, 2005 ▶).

The monochromatic X-ray beam then passes through another integral shutter assembly into the third experimental station, known as the Nanoprobe end-station, designated 26-ID-C, containing a Si_3_N_4_ exit window for the X-ray beam, a set of beam filters that can be used to attenuate the beam, and a final set of beam size defining slits as the final components of the beamline prior to the Hard X-ray Nanoprobe, which is located at the end of the beamline, 75 m from the X-ray source.

## 3. The Hard X-ray Nanoprobe

**Figure 5:**
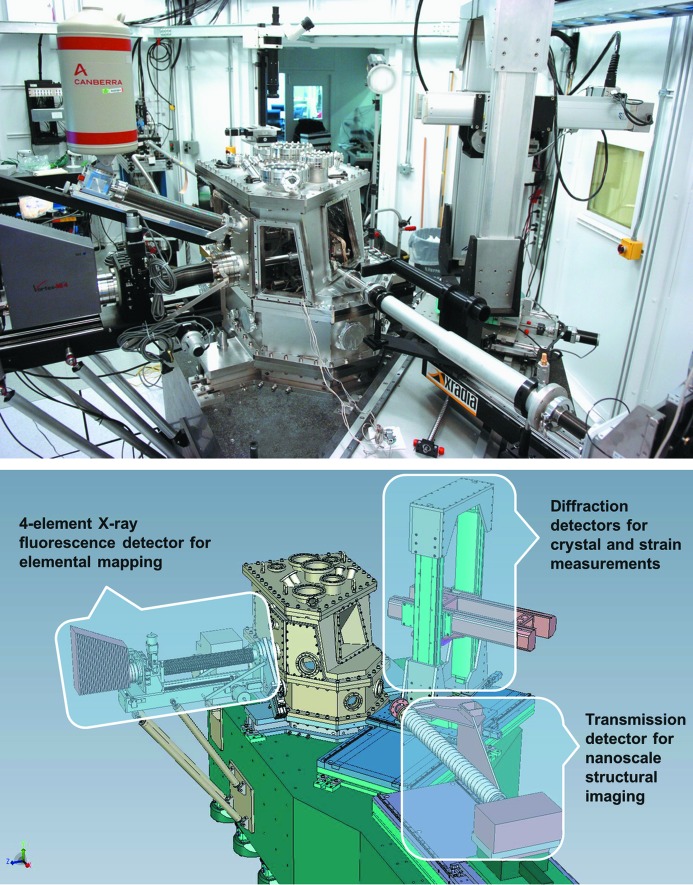
Photograph and schematic drawing of the Hard X-ray Nanoprobe showing the three detection modes used for nanoscale imaging.

**Figure 6:**
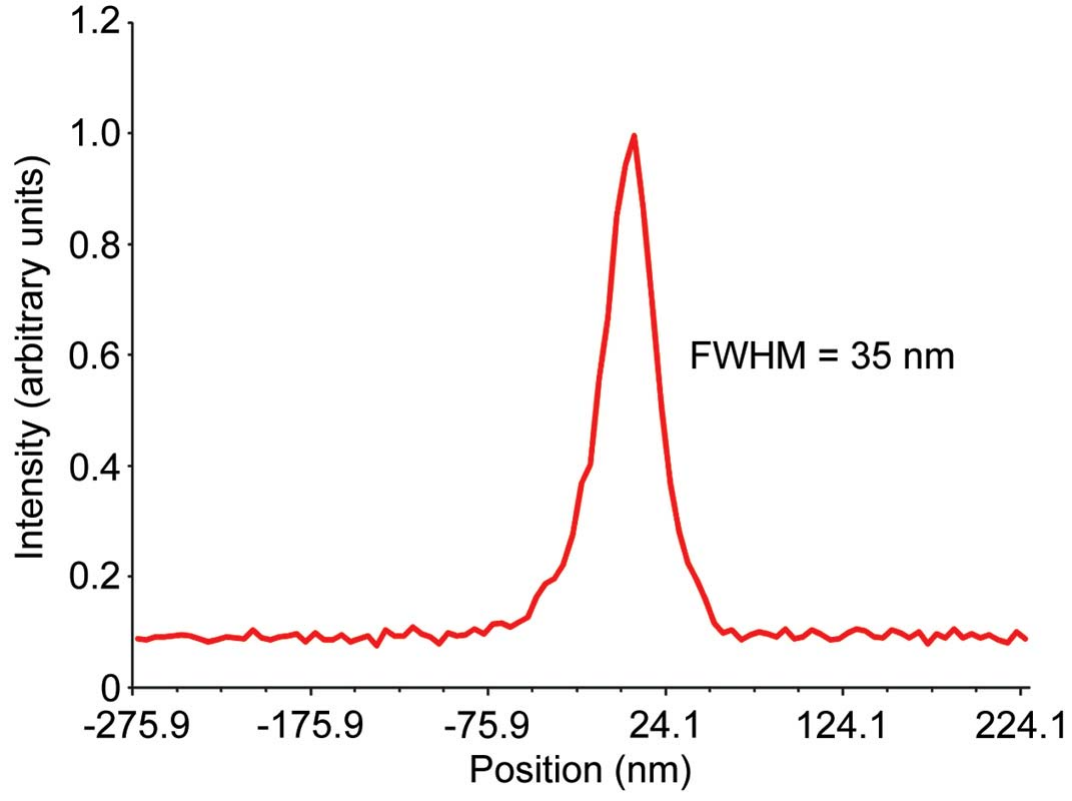
Line scan of test object showing scanning resolution system performance. Zone plate used for scanning, *D* = 133 µm and *dr* *N* = 24 nm (Xradia ZP 24-133).

The Hard X-ray Nanoprobe, shown in Fig. 5 ▶, is a combination scanning probe and full-field imaging microscope that incorporates fluorescence mapping, nanodiffraction and transmission imaging with absorption and phase contrast (Shu *et al.*, 2005 ▶, 2007 ▶). A detailed description of the design and performance of the Hard X-ray Nanoprobe is provided in an additional publication (Winarski *et al.*, 2012 ▶). For scanning probe experiments the coherent portion of the X-ray beam that has been prepared by the upstream optical components is directed onto a Fresnel zone plate which focuses the X-rays onto the sample (Kirz, 1974 ▶; Feng *et al.*, 2007 ▶). The Nanoprobe can incorporate several zone plates with different focal lengths to cover the 3–30 keV range of the beamline. The focal length of a zone plate depends on the incident photon energy according to the following relation,
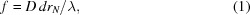
where *f* is the focal length, *D* is the outside diameter of the zone plate, *dr*_*N*_ is the width of the outermost zones of the zone plate, and λ is the wavelength of the incident X-ray beam. For 10 keV X-rays, for a typical zone plate used for scanning with an outside diameter of 133 µm and an outermost zone width of 24 nm, the focus is located 25.75 mm from the zone plate. This long focal distance allows for a large angle of acceptance for X-ray fluorescence detection, as well as the ability to create sizable environmental cells for sample measurements. With these zone plates we have measured a focused beam flux of 1.8 × 10^9^ photons s^−1^ at 9.75 keV. The focused resolution provided by the zone plate is related to the width of the outermost zones of the zone plate. For zone plates with an outermost zone width of 24 nm, the achievable resolution is 29.3 nm. To date, as shown in Fig. 6 ▶, we have been able to achieve a repeatable sub-40 nm resolution which includes vibration contributions to the system from floor oscillations, thermal fluctuations and other sources of acoustic noise around the beamline.

Accuracy of the laser feedback system is on the order of 4 nm peak to peak (this contributes 1.4 nm RMS in quadrature to the apparent beam size) over short time intervals. We have measured a positional stability of between 2 and 5 nm h^−1^ of uncontrolled drift over long scans depending on the amount of equilibration time prior to the start of a scan. In general, 50 nm step and settle scans are accurate to within a pixel over a 12 h-long scan after sufficient instrument equilibration time (2–3 h).

To map the elemental composition of a specimen in fluorescence mode, or nano-crystalline properties in diffraction mode, the zone plate focus is raster scanned across the sample. In order to accurately scan the sample and compensate for any vibrations that might affect the measurement, the Nanoprobe incorporates a laser measurement system that monitors the positions of the sample and scanning zone plate in three-dimensional space. These measurements are used to feed back position changes to a digital signal-processing system which controls piezoelectric-actuator-driven flexure stages underneath the scanning zone plate in order to maintain the proper zone plate to sample orientation (Shu *et al.*, 2006 ▶, 2007 ▶).

Fluorescence mapping utilizes a four-element silicon drift diode detection system (SII Nanotechnology, USA; Vortex-ME4) which is located perpendicular to the X-ray beam direction. Diffraction measurements can employ a number of detectors (Photometrics Coolsnap K4, Princeton Instruments PIXIS-XF 1024F, and Rayonix SX-165) which are located behind the sample, and positioned to reproduce goniometer accuracy in a horizontally diffracting geometry. The available diffraction volume [50° vertical (γ direction) by 65° horizontal (2θ direction)] requires that prior knowledge of diffraction planes in the sample is required for proper alignment. A single-element silicon drift diode detector (SII Nanotechnology, USA; Vortex-EM) mounted opposite and above the four-element detector is used to aid sample alignment in the diffraction detection alignment geometry.

**Figure 7:**
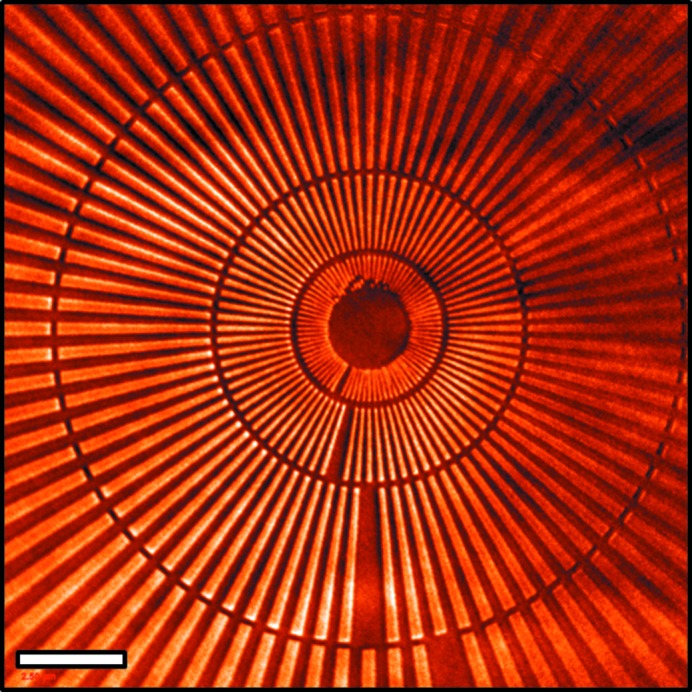
Full-field image of spoked test pattern. Inner spokes are 24 nm in width. Phasing effects in the image are due to protective film encapsulation of the test pattern and partial coherence of the illumination. The scale bar is 2.5 µm.

For full-field imaging and nanotomography the X-ray beam is directed onto a capillary condenser which focuses the X-rays onto the sample (Zeng *et al.*, 2008 ▶). X-rays diffracted by the sample are collected by a zone plate which magnifies and focuses the image onto imaging optics and a CCD camera located at the end of the beamline (Tkachuk *et al.*, 2007 ▶). This instrument is sensitive to both absorption contrast and Zernike phase contrast (Burch, 1934 ▶; Zernike, 1935 ▶) with the addition of a phase ring after the zone plate. Fig. 7 ▶ illustrates the performance of the nanoprobe in full-field mode using a gold spoked test pattern. The 24 nm features near the center of the pattern are visible. Nanotomographic images are obtained by combining this technique with a precision rotation stage. Images taken at regular angular intervals over a total rotation of 180° are aligned and reconstructed to produce a three-dimensional representation of a sample’s internal structural features.

## 4. Conclusion

The Hard X-ray Nanoprobe Beamline is a precision platform for scanning probe and full-field microscopy with 3–30 keV X-rays. The combination of high-stability X-ray optics and precision motion sensing and control enables detailed studies of the internal features of samples with a resolution approaching 30 nm. The Hard X-ray Nanoprobe is a combination scanning probe and full-field imaging microscope for fluorescence mapping, nanodiffraction and transmission imaging. Future improvements in X-ray optics fabrication and precision sensing and motion control will be incorporated into the beamline to enable imaging at a resolution approaching 10 nm.

## 5. Facility Access

The Hard X-ray Nanoprobe Beamline is jointly operated by Argonne National Laboratory’s Center for Nanoscale Materials and the Advanced Photon Source. The beamline is open to academia, industry, government agencies and research institutes worldwide for scientific investigations in the area of nanoscience. Access is obtained *via* brief peer-reviewed proposals with no charge for users who intend to publish their results. Prospective users are encouraged to contact staff members to learn more about the science and capabilities of the beamline. More information can be found here: http://www.cnm.anl.gov/users/index.html or http://www.aps.anl.gov/Users/Prospective/.
